# Bipartite Medial Cuneiform: Case Report and Retrospective Review of 1000 Magnetic Resonance (MR) Imaging Studies

**DOI:** 10.1155/2014/130979

**Published:** 2014-01-23

**Authors:** Geraldine H. Chang, Eric Y. Chang, Christine B. Chung, Donald L. Resnick

**Affiliations:** ^1^Department of Radiology, University of California (UCSD), San Diego, CA 92103, USA; ^2^Department of Radiology, VA San Diego Healthcare System, San Diego, CA 92161, USA

## Abstract

*Objective*. To present a unique case report of a Lisfranc fracture in a patient with a bipartite medial cuneiform and to evaluate the prevalence of the bipartite medial cuneiform in a retrospective review of 1000 magnetic resonance (MR) imaging studies of the foot. *Materials and Methods*. Case report followed by a retrospective review of 1000 MR imaging studies of the foot for the presence or absence of a bipartite medial cuneiform. *Results*. The incidence of the bipartite medial cuneiform is 0.1%. *Conclusion*. A bipartite medial cuneiform is a rare finding but one with both clinical and surgical implications.

## 1. Introduction

The bipartite medial cuneiform is a rare tarsal developmental variant at the Lisfranc joint that was first described in 1942 [1, 2]. Prior case reports have described how this rare anomaly could impact clinical care, either as a source of unexplained chronic midfoot pain or as a potential for misdiagnosis as a fracture in the setting of midfoot trauma [3–6].

We present a case report of a Lisfranc fracture in a patient with a bipartite medial cuneiform, an association that has not yet been described in the literature. Our case furthered exemplifies how knowledge of the bipartite medial cuneiform plays a role in both conservative and surgical treatment. Additionally, as we have encountered this anomaly only very rarely, we sought to determine the prevalence of the bipartite medial cuneiform with a retrospective review of 1,000 consecutive magnetic resonance (MR) imaging studies.

## 2. Materials/Method

The clinical and imaging findings of a patient with a Lisfranc fracture with a bipartite medial cuneiform were reviewed. This case led to a retrospective, cross-sectional, observation study that was approved by the Institutional Review Board with waiver of informed consent. One thousand MR imaging scans of the foot that were performed at our institution from January of 2007 to February of 2013 were reviewed. The typical MR imaging protocol included axial T1, axial proton density (fat sat), sagittal T1, sagittal T2 fast spin echo (fat sat), coronal T1, coronal T2 fast spin echo (fat sat) and coronal proton density (fat sat) sequences. Patients were selected on the basis of availability of MR imaging scans for review. Exclusion criteria were lack of available images (off-site storage) or artifacts due to excessive motion or metal. The findings in the case report were reviewed in consensus by a radiology resident (Geraldine Chang) and fellowship trained musculoskeletal radiologist (Eric Y. Chang, two years of experience) and thereafter the remainder of the MR images in the retrospective review were reviewed by the radiology resident (Geraldine Chang). Age and biological sex were also recorded.

## 3. Results

### 3.1. Case Report

A 31-year-old man presented to the emergency department after a skateboarding accident. The patient stated that he twisted his left foot and struck the dorsum of the foot against a curb. Pain was localized to his midfoot and worsened with weight bearing. Patient also reported a feeling of foot instability.

Physical examination revealed ecchymosis and edema on the dorsum of the left foot. There was extreme tenderness throughout the Lisfranc articulation, but mostly in the region of the first and second rays. Overall, alignment of the foot was within normal limits. Range of motion of the ankle was preserved. Motor strength was decreased secondary to discomfort. Intact sensation and brisk capillary refill were also noted.

**Figure 1:**
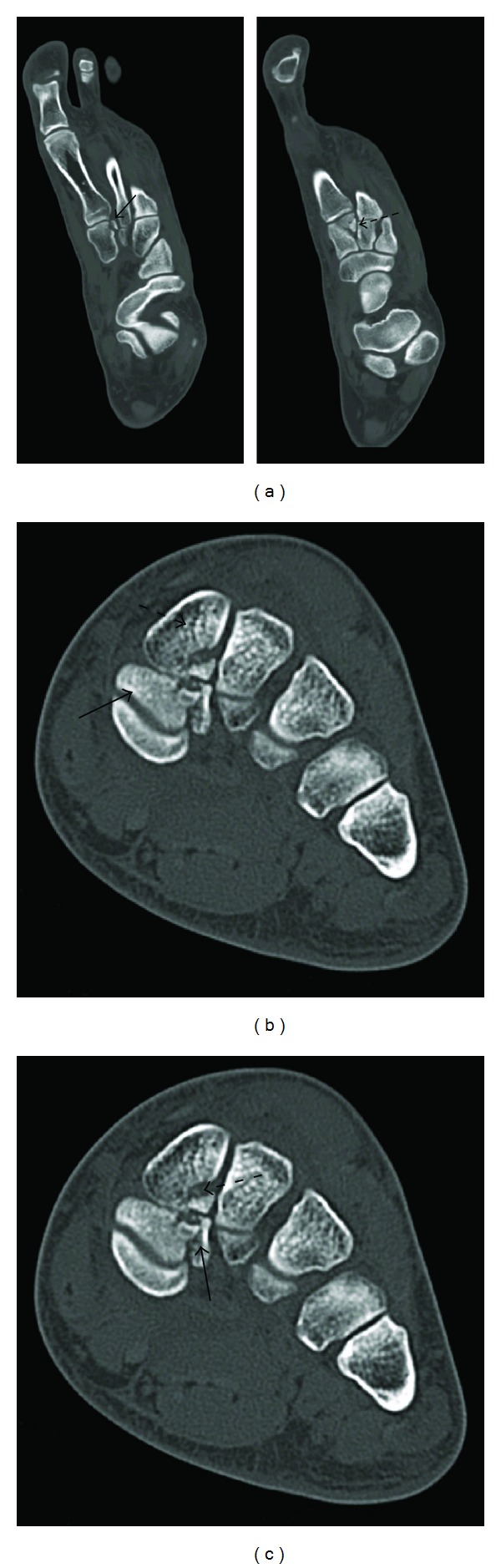
(a) Axial CT images at the level of the midfoot show a Lisfranc fracture of the medial cuneiform. The attachment of the plantar Lisfranc ligament (solid arrow) and the attachment of the Lisfranc ligament proper (dashed arrow) are demonstrated. (b) Coronal CT image at the level of the midfoot demonstrating the plantar (solid arrow) and dorsal (dashed arrow) segments of the bipartite medial cuneiform. (c) Coronal CT image at the level of the midfoot shows a Lisfranc fracture of the medial cuneiform. The attachment of the plantar Lisfranc ligament (solid arrow) and the attachment of the Lisfranc ligament proper (dashed arrow) are demonstrated.

**Figure 2:**
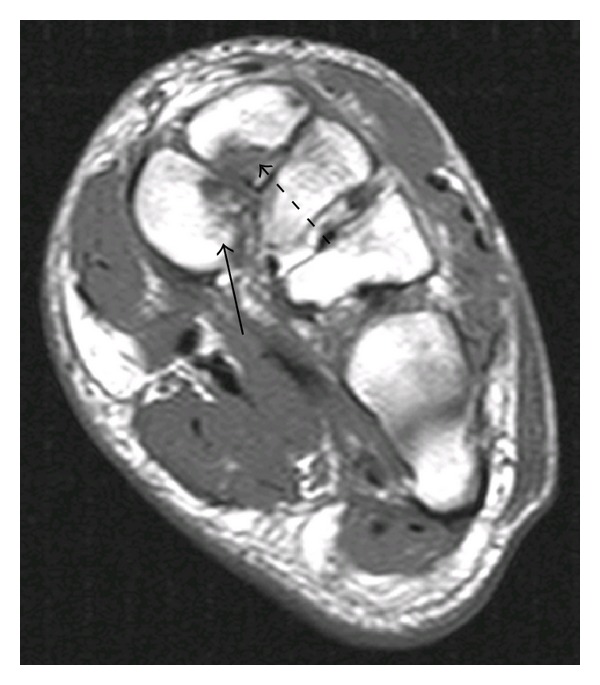
Coronal T1 MR image at the level of the midfoot shows a Lisfranc fracture of the medial cuneiform. The plantar Lisfranc ligament (solid arrow) and the Lisfranc ligament proper (dashed arrow) are demonstrated.

Radiographs, computed tomography (CT) images, and MR images (refer to Figures 1 and 2) revealed a Lisfranc injury in the setting of a bipartite medial cuneiform. MR images demonstrated the attachment site of the plantar Lisfranc ligament to the plantar segment of the medial cuneiform and the attachment site of the Lisfranc ligament proper and dorsal Lisfranc ligament to the dorsal segment of the medial cuneiform. Orthopedic consultation was obtained and patient underwent operative exploration, which demonstrated a dislocation of the first and second tarsometatarsal joints and a comminuted fracture of the medial and intermediate cuneiforms. Patient underwent open treatment with internal fixation.

This case report led to a retrospective review of 1000 MR images of the foot to evaluate the frequency of this anomaly. Subjects' age ranged from 17 to 92 years with a mean age of 45 years. There were 803 male and 197 female subjects. There was a 0.1% incidence of the bipartite medial cuneiform based on our review of 1000 MR imaging studies of the ankle.

## 4. Discussion

The bipartite medial cuneiform is a rare tarsal developmental variant at the Lisfranc joint [1, 2]. Differentiating this osseous variation from a fracture is important to both clinicians and radiologists. In addition, the bipartite medial cuneiform can be a potential source for nontraumatic or traumatic midfoot pain, and knowledge of this entity can play a role in both conservative and surgical treatment. A previous anthropologic population study in 1942 found this anomaly to occur in approximately 0.3% of persons [2]. A case series and literature review by Elias et al. described the MR imaging features of the bipartite medial cuneiform [3]. To our knowledge, however, our case report is the first to show a Lisfranc fracture in the setting of a bipartite medial cuneiform. These findings prompted a retrospective review to determine the frequency of the bipartite medial cuneiform based on MR imaging.

The bipartition of the medial cuneiform is a malsegmentation defect of the foot characterized by separation of the normal cuneiform into dorsal and plantar segments due to the presence of two primary ossification centers [1, 3, 6–9]. It is the failure of fusion of these two ossicles that results in a bipartite medial configuration. The ossification process in this bone begins in the second to third year of life [4, 7–9]. In many cases, these segments are held together by means of a cartilaginous or fibrocartilaginous bridge.

In this anomaly, the cuneiform bone is divided horizontally by a synchondrosis, partitioning the medial cuneiform into plantar and dorsal segments. The plantar ossicle is typically larger than the dorsal ossicle [2, 3]. The tibialis anterior tendon attaches to the proximal superomedial aspect of the dorsal segment. The posterior tibialis tendon attaches to the distal inferolateral portion of the plantar segment. The peroneus longus tendon attaches to the proximal inferomedial and distal inferolateral portions of the plantar segment. The Lisfranc ligament proper (interosseous portion) and the dorsal Lisfranc ligament extend from the dorsal segment of the bipartite medial cuneiform to insert into the base of the second metatarsal. The plantar Lisfranc ligament extends from the plantar segment of the bipartite medial cuneiform [3, 10].

Based on our review of the literature, the bipartite medial cuneiform is generally an incidental finding but one that rarely can be symptomatic [5, 6, 10]. Chiodo et al. reported a patient who failed conservative treatment, demonstrating that surgical excision may be indicated [5]. Therefore, it is important for radiologists and clinicians to keep bipartition of the bone in mind when evaluating acute or chronic midfoot pain.

In the setting of trauma, it is important to differentiate a bipartite medial cuneiform from a fracture [11]. The bipartite articulation is best visualized on a 30-degree external oblique radiograph of the foot. The site of partition should demonstrate smooth, well corticated margins, and the two portions of the bipartite cuneiform together are larger than the expected normal or fractured medial cuneiform. In addition, all cases of a bipartite medial cuneiform demonstrate bipartition along the long axis of the foot, and this orientation is uncommon for a fracture. Furthermore, with MR imaging, an asymptomatic bipartite medial cuneiform should not have been associated with bone marrow edema. Due to the well-defined joint space between the base of the first metatarsal and the distal portion of the medial bipartite cuneiform as well as between the two bipartite portions, a “E” joint space configuration is demonstrated on MR imaging in the sagittal plane, the so-called E-sign [3, 12].

We are the first to report a Lisfranc fracture in the setting of this anomaly. Our patient had both CT and MR imaging. The Lisfranc ligament proper and the plantar Lisfranc ligament attached to different portions of the bipartitioned bone. In our case, both ossicles were fractured and the patient underwent open reduction and internal fixation.

To our knowledge, our series is the only one to date dealing with the frequency of the bipartite medial cuneiform in persons undergoing MR imaging. In our retrospective review of 1000 patients, we found a 0.1% incidence, which is lower than that reported in anthropological studies.

There are limitations to our study. Our subjects included an uneven distribution of male and female subjects. In addition, the age range of our subject population was 17–92 with the exclusion of the pediatric population. Further studies that include a more diverse patient population with more female and pediatric subjects are required to validate our findings. However, the reported incidence in the literature of a bipartite medial cuneiform bone has ranged from 0.3% to 2.4% in cadaveric studies [3]. Our study confirms that the bipartite medial cuneiform is, in fact, very rare.
